# Postoperative radiotherapy might be a risk factor for second primary lung cancer: A population-based study

**DOI:** 10.3389/fonc.2022.918137

**Published:** 2022-10-06

**Authors:** You Mo, Minxin Chen, Meng Wu, Dawei Chen, Jinming Yu

**Affiliations:** ^1^ Department of Cardiovascular Medicine, The First Affiliated Hospital of Shantou University Medical College, Shantou, China; ^2^ Department of Radiation Oncology, Shandong Cancer Hospital and Institute, Shandong First Medical University and Shandong Academy of Medical Sciences, Jinan, China; ^3^ Department of Oncology, The Affiliated Hospital of Southwest Medical University, Luzhou, China

**Keywords:** initial primary lung cancer, secondary primary lung cancer, standardized incidence ratio (SIR), postoperative radiotherapy, overall survival (OS), cancer-specific survival (CSS)

## Abstract

**Background:**

Surgery is the main curative therapeutic strategy for patients with initial primary lung cancer (IPLC). Most international guidelines recommend regular follow-ups after discharge to monitor patients for tumor recurrence and metastasis. As the overall survival (OS) in patients with lung cancer improves, their risk of secondary primary lung cancer (SPLC) increases. Previous studies on such patients lack separate assessment of different survival outcomes and evaluation of high-risk factors for SPLC. Therefore, we aimed to determine the correlation between high-risk factors and causes of death in patients with SPLC, based on the Surveillance, Epidemiology, and End Results (SEER) database.

**Methods:**

We screened the SEER database for patients with IPLC and SPLC from 2004 to 2015 and included only patients who underwent surgery since the IPLC and in whom the cancer was pathologically verified of an International Classification of Diseases grade of 0-3 and to be non-small-cell lung cancer. The standardized incidence ratio (SIR) was calculated between variables and SPLC. Multivariable Cox proportional-hazards regression analyses were conducted to calculate the correlation of different variables with overall survival (OS) and cancer-specific survival (CSS). A competing-risk model was conducted for SPLC. The effect of baseline bias on survival outcomes by performing propensity score matching analysis in a 1: 6 ratio (SPLC: IPLC).

**Results:**

For patients aged 0-49 years, the overall SIR was higher in older patients, reaching a maximum of 27.74 in those aged 40-49 years, and at 11.63 in patients aged 50-59 years. The overall SIR was higher for patients who were more recently diagnosed with IPLC and increased with time after diagnosis. Male sex, SPLC (hazard ratio, 1.6173; 95% confidence interval, 1.5505-1.6869; *P* < 0.001), cancer grade III or IV, lower lobe of the lung, advanced stage and postoperative radiotherapy (PORT) were independently detrimental to OS. In terms of CSS, PORT was a high-risk factor.

**Conclusions:**

Postoperative radiotherapy is a risk factor for second primary lung cancer and detrimental to overall and cancer-specific survival in patients who had initial primary lung cancer. These data support the need for life-long follow-up of patients who undergo treatment for IPLC to screen for SPLC.

## Introduction

Lung cancer is one of the main causes of cancer-related deaths worldwide (1). Although various therapies have been used for local control, reduction of recurrence, and palliative care, including targeted therapy (2), immunotherapy (3), and radiotherapy, surgery remains the most important curative treatment. Although treatment modalities vary, most international guidelines recommend regular follow-up and chest computed tomography after discharge; however, the duration and frequency of optimal follow-up remains unclear. The main focus in the follow-up of patients with initial primary lung cancer (IPLC) after discharge is monitoring of recurrence and evaluation of treatment effectiveness. The occurrence of second primary lung cancer (SPLC) requires careful attention as the expected overall survival (OS) of patients with lung cancer is improving, which is accompanied by increased risk of SPLC in the patient population (4, 5).

Compared to the population with no lung cancer history, the risk of developing SPLC among patients who have had IPLC is the highest in the first year after treatment and remains high at 10 years (6). However, the current diagnostic criteria for SPLC are not standardized. There is also a lack of consensus on the cause of death and high-risk factors in patients diagnosed with SPLC. Many research teams have explored these issues. For example, one team developed a risk prediction model based on the metabolomic profiles of 82 SPLC cases and 82 frequency-matched IPLC controls; they proposed the use of an untargeted metabolomics approach to screen patients who have had IPLC and identify those at a high risk of SPLC (7). Smoking pack-years and smoking intensity have been repeatedly mentioned as risk factors for SPLC (8, 9). However, to our knowledge, the different causes of death have not been explored in patients with SPLC, despite probable differences in risk factors influencing cancer-specific survival (CSS), cardiovascular-related death, and chronic obstructive pulmonary disease (COPD)-related death. Such differences have been confirmed in unpublished studies by our research group. Moreover, for patients who develop SPLC, there is a need to explore whether the two primary tumors are completely independent events and whether the same risk factors have similar effects on both.

Therefore, we conducted a study in which we separately analyzed the different survival outcomes, with the aim of determining the correlation between high-risk factors and causes of death in patients with SPLC based on the Surveillance, Epidemiology, and End Results (SEER) database. We hope that our findings will help improve screening of the high-risk population for timely treatment and provide guidance for clinicians in monitoring the patients’ condition during regular follow-ups.

## Materials and methods

### Patient selection

Within Surveillance, Epidemiology and End Results (SEER)-18 registries, cancer patients who were diagnosed at 2001-2015 had been reviewed. We screened the SEER database for patients with initial (n = 589,722) and second (n = 11,105) primary cancers in the lungs and bronchi. Among these, we included only patients who underwent surgery since the IPLC was diagnosed and those in whom the cancer was pathologically verified to have an International Classification of Diseases grade of 0-3 and to be non-small-cell lung cancer. We excluded patients for whom the cancer grade, stage, laterality, and/or radiation treatment status were unknown. SPLC was defined by the well-established Martini and Melamed criteria: if the new tumor was diagnosed over 2 years after the IPLC diagnosis, or the histology of new tumor is different from the histology of IPLC developed within 2 years (9, 10). Considering the definition of SPLC, we decided to also exclude patients with a survival time of <24 months. For analysis in this study, we treated IPLC and SPLC in the same patient as independent events.

### Ethics statement

This study was based on the SEER database and was conducted in compliance with the tenets of the Declaration of Helsinki. Permission was obtained to access the files containing the SEER program research data (reference number: 15388-Nov2020). Informed consent was not required because the patients were not personally identifiable.

### Statistical analysis

Statistical analyses were performed using Stata MP 16 (StataCorp LLC) and R 4.0 software and R package ggforest and ggsurvplot to visualizes the data were used to generate graphs. The variables analyzed for all patients were race, sex, age, primary site, tumor grade, laterality, tumor histology, staging, radiotherapy status, chemotherapy status, and record. The observation endpoints of the study were OS, CSS, cardiovascular-related death, and COPD-related death, all extracted from the SEER database. Observed/Expected (O/E) ratios (with 95% confidence intervals (CI)) of second primary malignancies (SPC) among these cases were calculated by comparison to the age-adjusted cancer incidence in the general population through SEER*stat software (version 8.3.8) for the patients’ cohort as well as for patients’ subgroups that were defined according to clinicopathological characteristics (age, race, sex, treatment, and SEER summary stage). The standardized incidence ratio (SIR) was used to express the observed disease incidence of our cohort relative to the expected disease incidence in the general population. Chi-square tests of independence (for categorical data) and Student’s t-tests (for continuous data) were used to compare baseline characteristics between the IPLC and SPLC groups. We attempted to minimize the effects of latent differences by performing propensity score matching analysis. Matching was performed for race, age, and cancer stage and histology. We stratified the baseline characteristics of matched patients according to the occurrence of SPLC. Univariate and multivariable Cox proportional-hazards regression analyses were used to calculate the correlation between different variables and OS, CSS, cardiovascular-related death, and COPD-related death. The cumulative incidence of SPLC was calculated with a competing-risk model. All statistical tests were two-sided, and statistical significance was set at *P* < 0.05.

## Results

### Patient demographics

**Figure 1:**
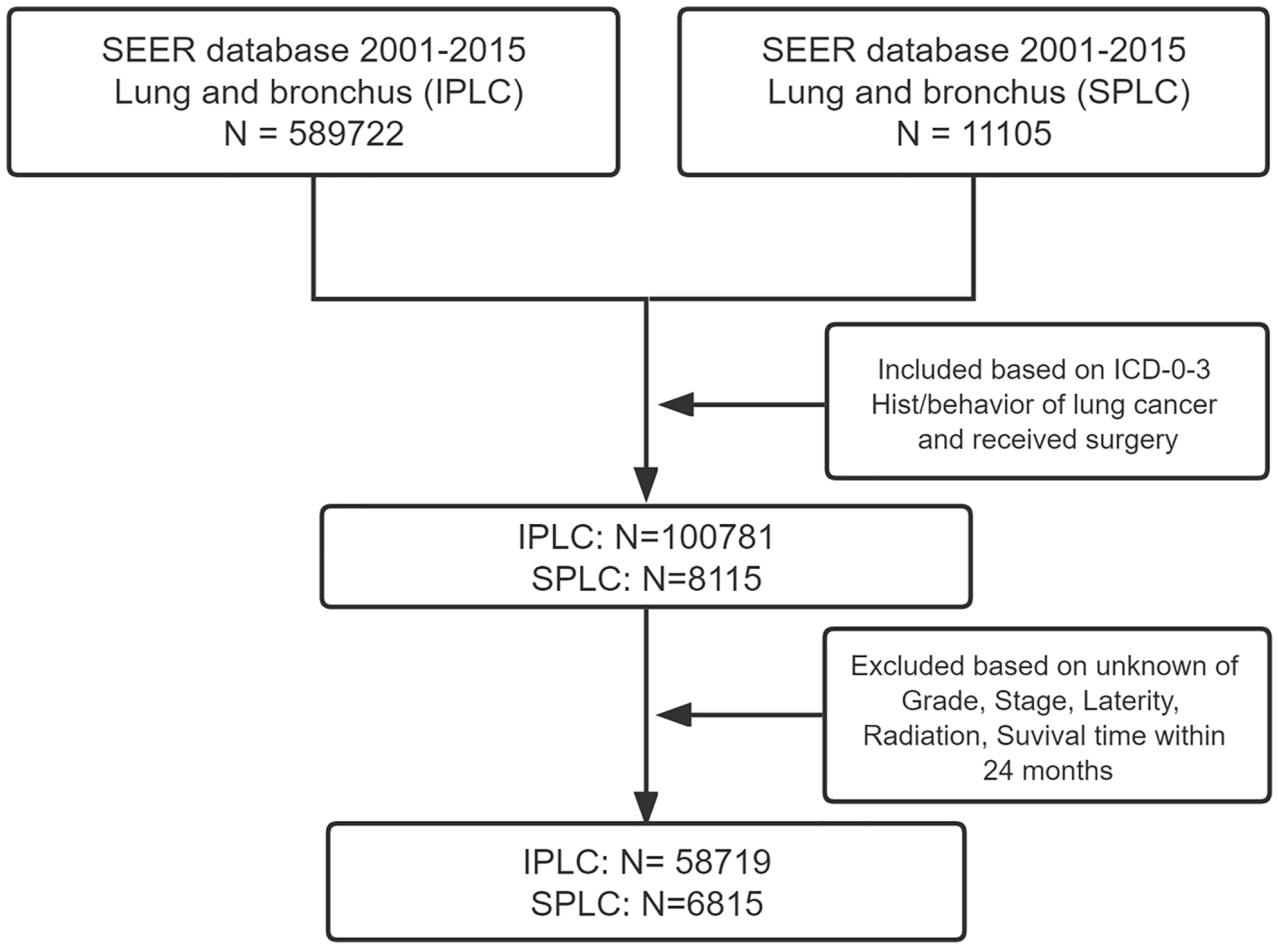
Selection strategy of patients.

After applying the inclusion and exclusion criteria, a sample of 91,883 patients with IPLC and 7,117 with SPLC were included for the analysis. After rigorously screening, we included 58,719 patients in the IPLC group and 6,815 in the SPLC group (**Figure 1**). We matched patients with IPLC to those with SPLC in a 6: 1 ratio because of the disparity in group sizes. Baseline characteristics of included patients in the current study were detailed in **Table 1**. The majority of included patients in IPLC groups were females (58.0%), white race (85.4%) and aged between 60 and 69 (39.1%). 29126 patients (93.9%) who did not receive postoperative radiotherapy (PORT) and 25381 patients (81.8%) who did not receive chemotherapy. Here, 94.6% of patients were in early stage of lung cancer. 58.1% of patients had Grade I or II, while 60.7% of tumor were located in upper lobe of lung. Those patients in SPLC groups were females (56.0%), white race (85.4%) and aged between 60 and 69 (39.2%). Here, 450 patients (7.2%) who received PORT and 1288 patients (20.6%) who received chemotherapy. The mean of interval time between IPLC and SPLC was 54.8 months. As summarized in **Table 1**, patient sex, cancer grade, PORT status, and chemotherapy status significantly differed between the groups.

**Table 1:** All Patients after propensity score matching, stratified by Second Primary Lung Cancer (SPLC) vs. Initial Primary Lung Cancer (IPLC).

	non-PORT	PORT	*P*-value
	(N=31013)	(N=6260)	
**Sex**			
Female	17997 (58.0%)	3504 (56.0%)	0.0028
Male	13016 (42.0%)	2756 (44.0%)	
**Age**			
0-49	1807 (5.8%)	322 (5.1%)	0.0517
50-59	6467 (20.9%)	1255 (20.0%)	
60-69	12127 (39.1%)	2453 (39.2%)	
70-79	9076 (29.3%)	1894 (30.3%)	
80+	1536 (5.0%)	336 (5.4%)	
**Race**			
White	26488 (85.4%)	5345 (85.4%)	0.517
Black	2682 (8.6%)	561 (9.0%)	
Others	1843 (5.9%)	354 (5.7%)	
**Primary**			
Upper lobe, lung	18810 (60.7%)	3811 (60.9%)	0.226
Lower lobe, lung	9487 (30.6%)	1891 (30.2%)	
Main bronchus	165 (0.5%)	31 (0.5%)	
Middle lobe, lung	1827 (5.9%)	352 (5.6%)	
Others	724 (2.3%)	175 (2.8%)	
**Grade**			
I and II	18024 (58.1%)	3325 (53.1%)	<0.001
III and IV	9668 (31.2%)	2318 (37.0%)	
Others	3321 (10.7%)	617 (9.9%)	
**Summary stage**			
Early stage	29330 (94.6%)	5927 (94.7%)	0.755
Advanced stage	1683 (5.4%)	333 (5.3%)	
**PORT**			
non-PORT	29126 (93.9%)	5810 (92.8%)	0.00112
PORT	1887 (6.1%)	450 (7.2%)	
**Chemotherapy**			
non-Chemotherapy	25381 (81.8%)	4972 (79.4%)	<0.001
Chemotherapy	5632 (18.2%)	1288 (20.6%)	

### SIR according to baseline characteristics

The whole patients’ cohort of SPLC as well as for patients’ subgroups were defined according to clinicopathological characteristics which were detailed in **Table 2**. The overall SIR was 4.68 compared to the general US population, which was significant and this means a high risk of SPLC. We firstly observed an increase in SIR of age at diagnosis, with 0-49 (27.74, 95% CI, 23.83-32.11), 50-59 (11.63, 95% CI, 10.98-12.31), 60-69 (6.44, 95% CI, 6.23-6.65), 70-79 (4.3, 95% CI, 4.18-4.42) and ≥80 (3.28, 95% CI, 3.14-3.43). Similarly, calendar year of diagnosis showed gradually changing trend, with 2000-2005 (2.28, 95% CI, 2.14-2.42), 2006-2010 (4.56, 95% CI, 4.41-4.71), 2011-2015 (6.05, 95% CI, 5.890-6.21). Moreover, SIR of SPLC was obviously higher with longer latency period, changing from 6-11 months (1.83, 95% CI, 1.70-1.97), 12-35 months (3.52, 95% CI, 3.39-3.65), 36-59 months(6.43, 95% CI, 6.20-6.67), 60-119 months (7.58, 95% CI, 7.35-7.82), ≥120 months (7.16, 95% CI, 6.73-7.6). When observed variable was laterality, left (5.24, 95% CI, 5.10-5.39) was higher than right (4.84, 95% CI, 4.72-4.96). Female showed higher SIR (5.83, 95% CI, 5.68-5.97) than male (4.18, 95% CI, 4.07-4.29). Consistent with consensus, tumor grade II and III held higher SIR, with I(5.17, 95% CI, 4.88-5.46), II (6.11, 95% CI, 5.92-6.31), III (5.55, 95% CI, 5.37-5.74), IV (54.89, 95% CI, 4.46-5.36). Considering of therapies, chemotherapy (4.55, 95% CI, 4.40-4.69) showed a lower SIR than non-Chemotherapy (5.11, 95% CI, 5.00-5.22).

**Table 2:** Standardized incidence ratios for second lung cancer risk in patients with lung cancer by characteristic.

			Standardised incidence rate ratio	
Characteristic	O	E	SIR (O/E)	(95% CI)
**Total**	13041.00	2787.63	4.68 *	4.60-4.76
**Age at diagnosis, years**				
0-49	179.00	6.45	27.74 *	23.83-32.11
50-59	1183.00	101.70	11.63 *	10.98-12.31
60-69	3646.00	566.24	6.44 *	6.23-6.65
70-79	4816.00	1119.91	4.30 *	4.18-4.42
80+	2017.00	614.43	3.28 *	3.14-3.43
**Calendar year of diagnosis**				
2000-2005	1018.00	447.21	2.28 *	2.14-2.42
2006-2010	3635.00	796.96	4.56 *	4.41-4.71
2011-2015	5838.00	965.00	6.05 *	5.890-6.21
**Latency period, months**				
6-11	739.00	403.61	1.83 *	1.70-1.97
12-35	3041.00	864.25	3.52 *	3.39-3.65
35-59	2946.00	457.98	6.43 *	6.20-6.67
60-119	4063.00	535.93	7.58 *	7.35-7.82
≥120	1052.00	146.96	7.16 *	6.73-7.6
**Race**				
White	10159.00	2070	4.91 *	4.81-5.00
Black	1108.00	23.00	4.76 *	4.48-5.05
Other	572.00	101.77	5.62 *	5.17-6.10
Unknown	2.00	3.91	0.51	0.06-1.85
**Laterality**				
Right	6621.00	1368.01	4.84 *	4.72-4.96
Left	5124.00	977.59	5.24 *	5.10-5.39
**Grade**				
I	1257.00	243.36	5.17 *	4.88-5.46
II	3858.00	631.01	6.11 *	5.92-6.31
III	3670.00	660.97	5.55 *	5.37-5.74
IV	462.00	94.41	4.89 *	4.46-5.36
Unknown	2594.00	778.99	3.33 *	3.320-3.46
**Sex**				
Male	5545.00	1328.04	4.18*	4.07-4.29
Female	6296.00	1080.70	5.83*	5.68-5.97
**RT**				
No	10528.00	2182.08	4.82 *	4.73-4.92
Prior to	245.00	34.29	7.14 *	6.28-8.10
PORT	1016.00	183.03	5.55 *	5.21-5.90
**Chemotherapy**				
No	8095.00	1584.54	5.11 *	5.00-5.22
Yes	3746.00	824.20	4.55 *	4.40-4.69

Excess risk is per 10,000.

Confidence intervals are 95%.

* *P*<0.05.

### Risk factors detrimental to OS in patients with lung cancer

**Figure 2:**
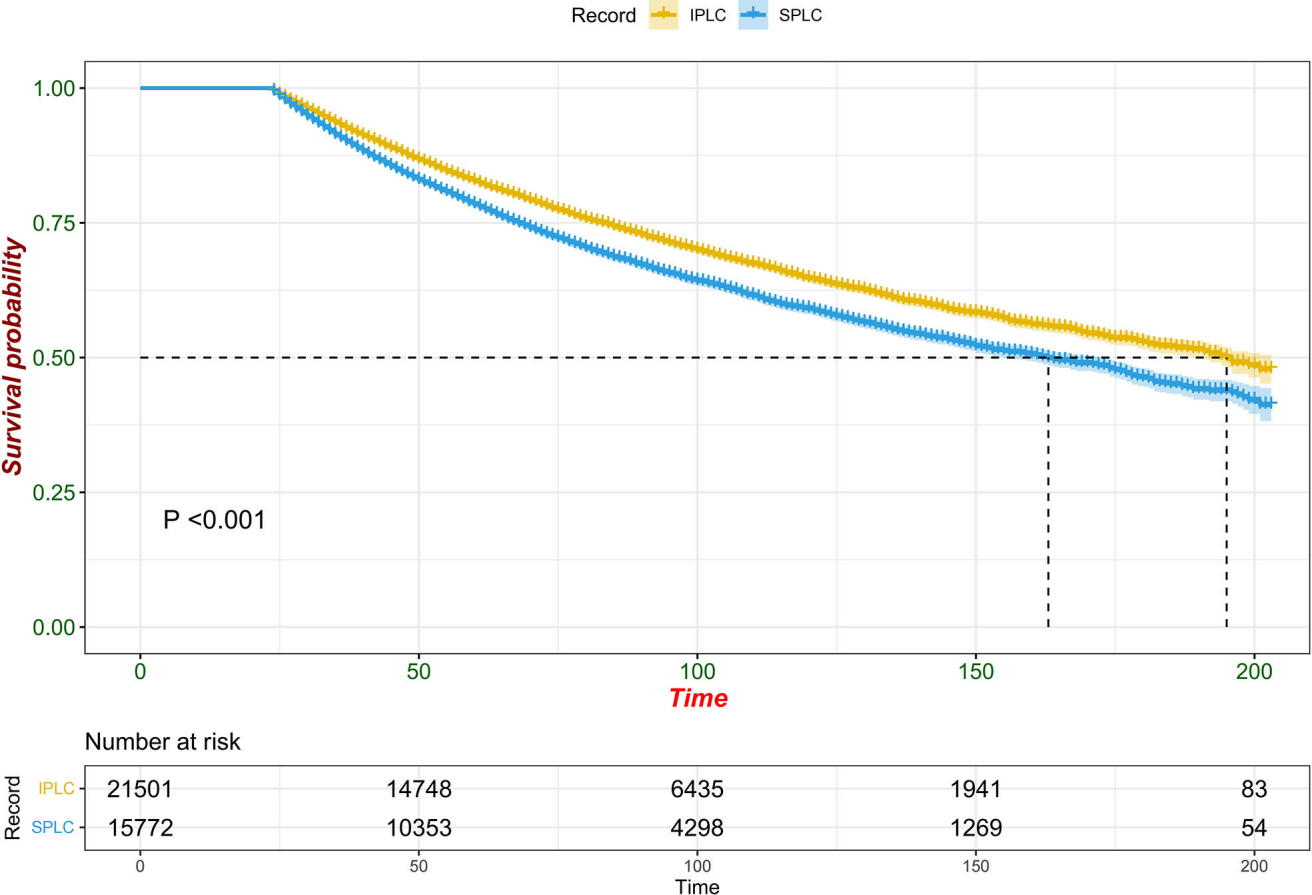
Forest plot of association between survival and SPLC diagnosis (vs. IPLC) in multivariable cox regression.

**Figure 3:**
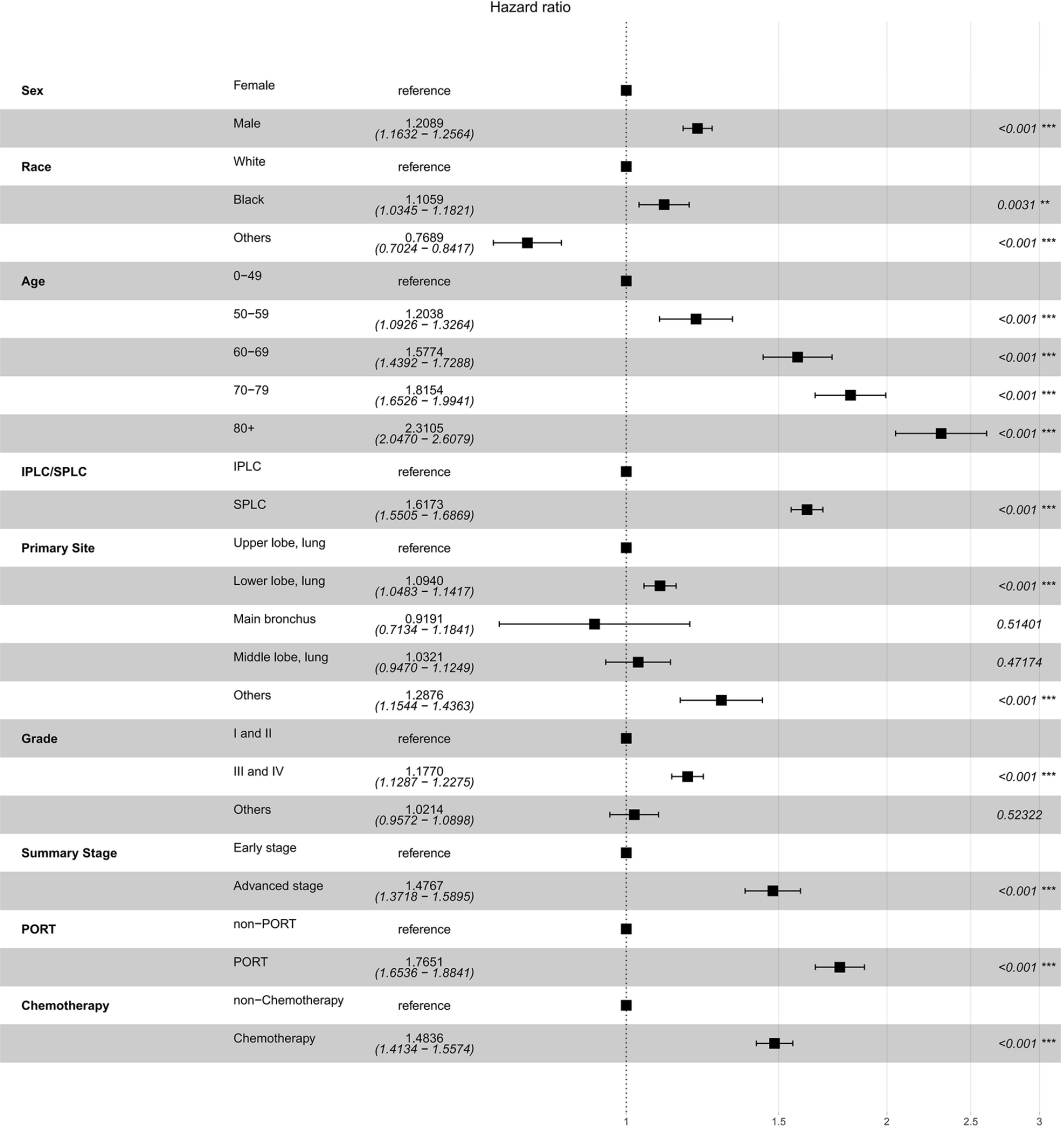
Adjusted survival curves for survival possibility among patients with SPLC vs. IPLC.

Since multiple variables of SPLC had higher SIR, we performed propensity score matching analysis in a 1: 6 ratio (SPLC: IPLC) to examine the influence to OS from primary lung cancer record and other variables. Adjusted survival curve showed the change in survival outcomes over time among patients with SPLC vs IPLC (HR, 1.6173; 95% CI, 1.5505-1.6869; *P* < 0.001) (**Figure 2**). According to the multivariable analysis, male sex (hazard ratio [HR], 1.2089; 95% CI, 1.1632-1.2564; *P* < 0.001), cancer grade III or IV (HR, 1.1770; 95% CI, 1.1287-1.2275; *P* < 0.001), lower lobe of the lung (HR, 1.0940; 95% CI, 1.0483-1.1417; *P* < 0.001), advanced stage (HR, 1.4767; 95% CI, 1.3718-1.5895; *P* < 0.001), PORT (HR, 1.7651; 95% CI, 1.6536-1.8841; *P* < 0.001), and chemotherapy (HR, 1.4836; 95% CI, 1.4134-1.5574; *P* < 0.001) were independently detrimental to OS. Furthermore, we noticed that the HR increased with age: 50-59 (HR, 1.5038; 95% CI, 1.0926-1.3264; *P* < 0.001), 60-69 (HR, 1.5774; 95% CI, 1.4392-1.7288; *P* < 0.001), 70-79 (HR, 1.8154; 95% CI, 1.6526-1.9941; *P* < 0.001) and ≥80 (HR, 2.3105; 95% CI, 2.0470-2.6079; *P* < 0.001) years (**Figure 3**).

### Detrimental and beneficial factors for different prognostic endpoints

**Figure 4:**
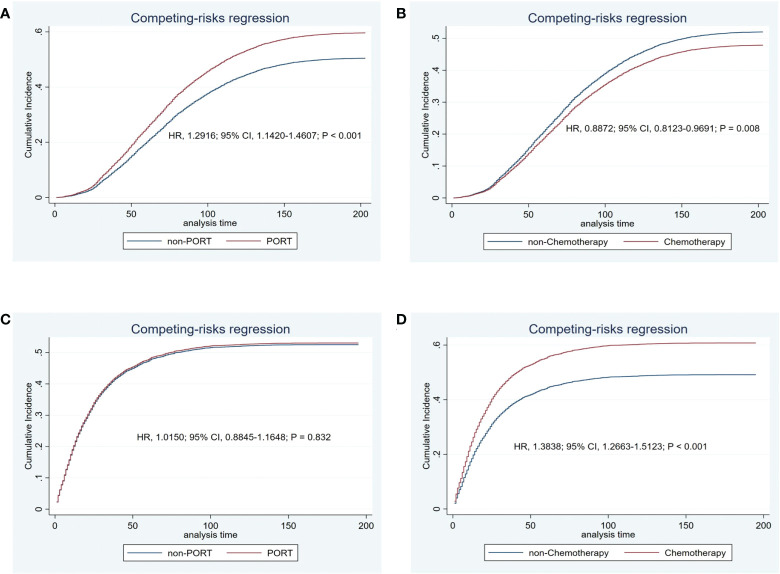
Cancer-specific cumulative incidence of estimates of patients with initial lung cancer by PORT (vs. non-PORT) **(A)** and chemotherapy (vs. non-chemotherapy) **(B)** and secondary lung cancer by PORT (vs. non-PORT) **(C)** and chemotherapy (vs. non-chemotherapy) **(D)**.

**Figure 5:**
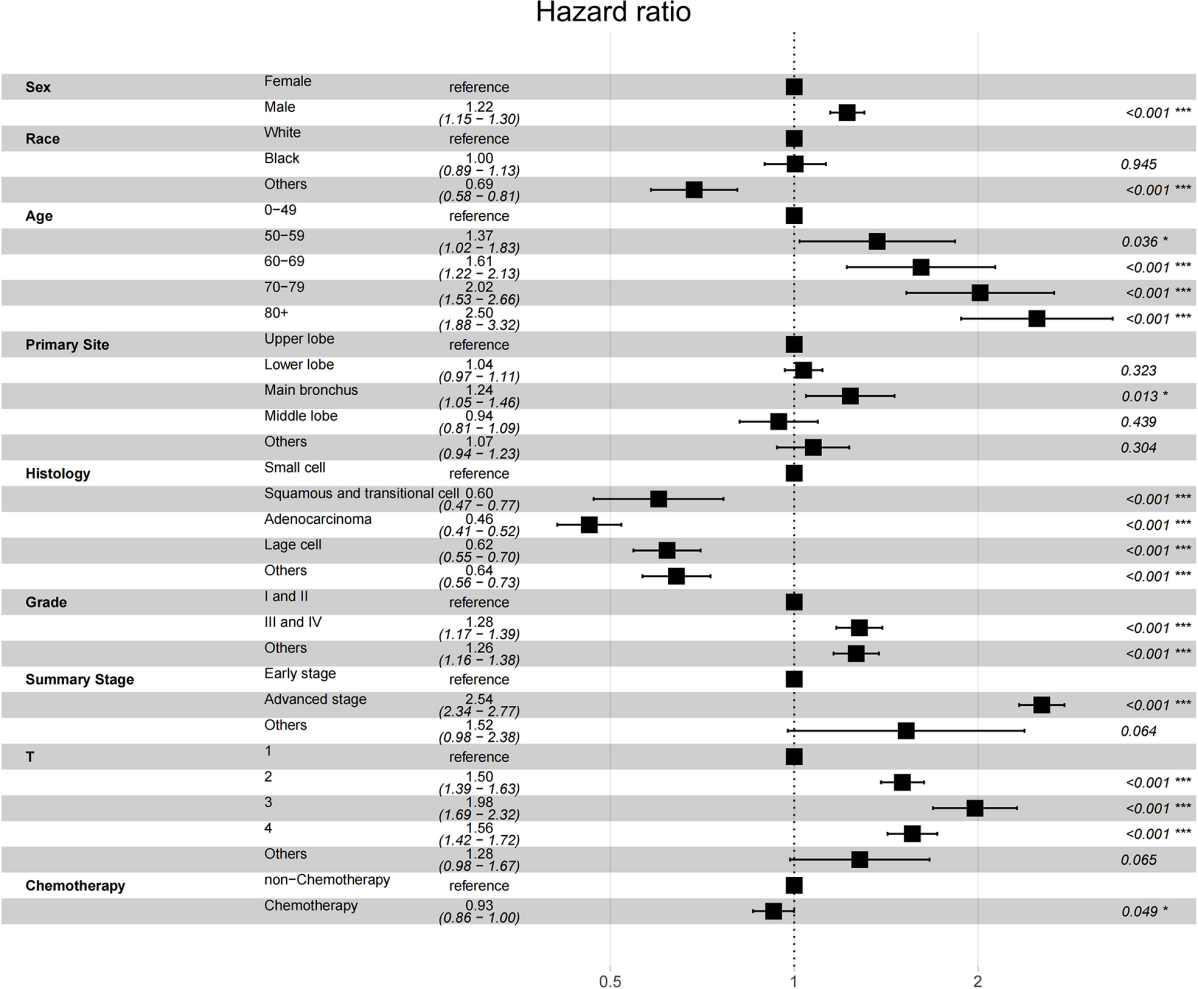
Forest plot of association between survival and multivariables in patients with SPLC diagnosis.

After finding that secondary lung cancer group was detrimental to OS, we tried to explore whether different variables played the same role in the two treatments for patients with secondary lung cancer group, especially the treatment modality. We divided patients who developed SPLC finally into initial lung cancer group and secondary lung cancer group by their primary lung cancer stage. In the initial lung cancer group, male sex (HR, 1.2177; 95% CI, 1.1404-1.3003; *P* < 0.001), older age (HR, 1.1031; 95% CI, 1.0641-1.1435; *P* < 0.001), PORT (HR, 1.2916; 95% CI, 1.1420-1.4607; *P* < 0.001), and adenocarcinoma (HR, 1.1071; 95% CI, 1.0613-1.1548; *P* < 0.001) were independently detrimental to CSS, while white race (HR, 0.9125; 95% CI, 0.8545-0.9745; *P* = 0.006) and chemotherapy (HR, 0.8872; 95% CI, 0.8123-0.9691; *P* = 0.008) were independently beneficial to CSS. Impact of PORT on death from lung cancer was further evaluated among different clinically defined subgroups (according to age at diagnosis, sex, race, grade, primary site, and summary stage). The result showed that female (HR, 1.264; 95% CI, 1.076-1.486; *P* = 0.004), white race (HR, 1.170; 95% CI, 1.031-1.328; *P* = 0.015), 50-59 years (HR, 1.330; 95% CI, 1.043-1.696; *P* = 0.022), 60-69 years (HR, 1.410; 95% CI, 1.177-1.690; *P* < 0.001), upper lobe (HR, 1.166; 95% CI, 1.022-1.329; *P* = 0.022), adenocarcinoma(HR, 1.317; 95% CI, 1.111-1.560; *P* = 0.001), tumor grade I or II(HR, 1.265; 95% CI, 1.051-1.522; *P* = 0.013), early stage(HR, 1.189; 95% CI, 1.050-1.347; *P* = 0.006) were prone to SPLC. In the secondary lung cancer group, older age (HR, 1.0974; 95% CI, 1.0527-1.1439; *P* < 0.001), cancer grade III or IV (HR, 1.1662; 95% CI, 1.1155-1.2193; *P* < 0.001), advanced stage (HR, 1.7984; 95% CI, 1.6307-1.9835; *P* < 0.001), and chemotherapy (HR, 1.3838; 95% CI, 1.2663-1.5123; *P* < 0.001) were independently detrimental to CSS while PORT showed no significant different (HR, 1.0150; 95% CI, 0.8845-1.1648; *P* = 0.832)(**Figure 4**). Furthermore, we examined the influence of chemotherapy to OS (HR, 0.9300; 95% CI, 0.8600-1.000; *P* = 0.049) (**Figure 5**). Lung cancer survivors had high risk of SPLC and PORT for IPLC might be detrimental for patients with SPLC. Meanwhile, considering of CSS, patients might benefit from chemotherapy who diagnosed IPLC, while benefit from chemotherapy who diagnosed SPLC when the endpoint was OS.

## Discussion

Patients who have undergone surgery for lung and bronchus cancer have a high risk of recurrence and death rate, even after complete resection (11). Therefore, follow-ups and regular monitoring after discharge are crucial. However, doctors normally pay more attention to IPLC than to the possibility of SPLC. As there is a lack of research on SPLC, we used the SEER database to explore high-risk factors for different causes of death in patients with SPLC. Our study suggested that lung cancer survivors have high risk of SPLC and PORT for IPLC may be detrimental with SPLC. The results may guide clinicians in treatment and disease monitoring in patients with primary lung cancer.

### SIR and high-risk variables

First, we notice that the stratified SIR is higher than the overall SIR of 4.68 in this table. We believe that it indicates that patients diagnosed with IPLC have a high tendency of developing SPLC. This may be ascribed to the unique structure of the lungs; they are composed of loose connective tissue. In simple terms, when a certain part of the lung develops a tumor, both it and other high-risk factors cause widespread changes in the lung tissue; the surrounding lung tissues share the same carcinogenic environment and undergo corresponding changes. Second, with the improvements in equipment and technology, such as low-dose computed tomography and other screening methods, detection of SPLC has improved (12). This may be why we observed an increase in SIR as the year of IPLC diagnosis approached the present. Third, young and middle-aged patients are expected to have a longer survival time than older patients upon diagnosis with IPLC, which is the likely reason why patients aged 0-49 had a higher SIR (27.74) than older patients. Specifically, the SIR at diagnosis seems to gradually increase up to a peak in the fifth decade of life. With advancing therapeutic, lung cancer survivors are rapidly increasing in number. Clinical trials, treatment guidelines and further research for SPLC are needed.

### PORT as a high-risk factor

From our results, the risk of SPLC may be even higher than previously thought. This risk seems to be underestimated by clinicians, as there have been few published studies on the risk factors for SPLC (13). In a previous study of patients in the SEER database, radiotherapy for IPLC seemingly did not affect the development of SPLC (14). Therefore, we explored the risk that PORT and chemotherapy poses in terms of different death outcomes as treatment for initial lung cancer group and secondary lung cancer group in those same patients. We used appropriate methods to adjust for competing risks and revealed that PORT for IPLC was detrimental to CSS for patients with SPLC.

Although PORT is performed with the aim of reducing the recurrence risk in patients with lung cancer, it is not indicated for every patient. For example, Leroy et al. suggested that patients who received postoperative thoracic radiotherapy were at higher risk than those who did not after complete non-small-cell lung cancer resection (15). In a study on patients with stage IIIA-N2 non-small-cell lung cancer, receiving 3D-conformal PORT was not associated with a higher disease-free survival rate than not receiving PORT. A higher proportion of SPLC(5%) was observed in the PORT group than in the control group(1%), which was inferior to cardiopulmonary. Patients with SPLC could not be excluded whether they were affected by PORT (16). which will require more detailed analyses. In a study on rectal cancer, the cumulative incidence of mortality was higher in patients who received radiotherapy than in those who did not, as they generally had more advanced disease, although death was a strong competitor for second primary malignancy in both patient groups (17). Moreover, epidemiological data suggest that most second solid cancers in patients who received radiation followed a linear dose response (18). The second cancer may be induced directly by the radiation beam passing through the normal tissue or by intermediate radiation products, resulting in the induction, deletion, or transcription of genes in normal cells (19). Compared to patients who did not undergo surgical resection, those who did were more likely to sustain damage to their normal cells upon irradiation. This indicates that any dose increase to the surrounding tissue may result in treatment-induced second cancer and a corresponding reduction in survival benefit. Radiation may induce second primary tumors not only in the target organ but also in other regions. There is a clear trend of a higher relative risk of second cancers in organs outside of the irradiated target compared to patients who were not irradiated (20–25). There is a need to study the mechanism by which PORT affects patient survival outcomes. Moreover, there is a clear need not only to improve radiotherapy techniques to minimize normal tissue doses but also to develop combination therapies. The addition of chemotherapy is widely recognized to have an additive effect in the development of second tumors (26). In our study, chemotherapy for IPLC seemed to benefit patients with SPLC in terms of CSS and cardiovascular-related death. It is unlikely that the results of this study regarding PORT were biased by chemotherapy, as PORT and chemotherapy had opposite effects on survival outcomes.

Our results suggest that there are several high-risk factors for the development of SPLC in patients who were treated for IPLC, most notably those receiving PORT for IPLC. Hence, treatment strategies may need to be changed for this population and regular follow-ups are vital. Thus, the emerging challenge in lung cancer treatment is to identify patients who will benefit from PORT or chemotherapy and who will derive no additional benefit or even have an increased risk of SPLC following such treatment.

Previous study has shown that tobacco smoking was a risk factor for SPLC among IPLC patients (8), but data on smoking status and specific radiotherapy techniques were not available in the SEER database and were not included in this analysis. In addition, one relevant question concerns the relationship between radiotherapy techniques in patients with SPLC on survival and how radiotherapy techniques contribute to survival differences. Future directions should aim to elucidate these relationships and mechanism.

## Conclusion

Following IPLC surgery, it is of utmost importance to monitor patients for the development of SPLC, as survival time for such patients is increasing. Multiple factors, including a longer time since and a younger age at IPLC diagnosis, yield a higher SIR for the development of SPLC. Furthermore, PORT for IPLC is significantly detrimental to CSS and OS.

## Data availability statement

The raw data supporting the conclusions of this article will be made available by the authors, without undue reservation.

## Author contributions

YM designed the work and wrote the manuscript. MC and MW analyzed and interpreted the patient data. DC was responsible for the management and coordination of the planning and execution of research activities. DC and JY revised the manuscript. JY was the major contributor in writing the manuscript. All authors contributed to the article and approved the submitted version.

## Funding

Natural Science Foundation of China, Grant/Award Number: 82172676; Science Foundation of Shandong, Grant/Award Number: ZR2020LZL016 and ZR2021YQ52; Foundation of Bethune Charitable, Grant/Award Number: 2021434953; Young Elite Scientist Sponsorship Program By Cast, Grant/Award Number: No. YESS20210137; Foundation of National Natural Science Foundation of China, Grant/Award Number: 81627901, 81972863 and 82030082.

## Conflict of interest

The authors declare that the research was conducted in the absence of any commercial or financial relationships that could be construed as a potential conflict of interest.

## Publisher’s note

All claims expressed in this article are solely those of the authors and do not necessarily represent those of their affiliated organizations, or those of the publisher, the editors and the reviewers. Any product that may be evaluated in this article, or claim that may be made by its manufacturer, is not guaranteed or endorsed by the publisher.

## Supplementary material

Supplementary file S1 Subgroup analysis between PORT and non-PORT group with IPLC.
